# Using point-of-care C-reactive protein to guide antibiotic prescribing for lower respiratory tract infections in elderly nursing home residents (UPCARE): study design of a cluster randomized controlled trial

**DOI:** 10.1186/s12913-020-5006-0

**Published:** 2020-02-27

**Authors:** Tjarda M. Boere, Laura W. van Buul, Rogier M. Hopstaken, Ruth B. Veenhuizen, Maurits W. van Tulder, Jochen W. L. Cals, Theo J. M. Verheij, Cees M. P. M. Hertogh

**Affiliations:** 1 Department of General Practice & Old Age Medicine, Amsterdam Public Health Research Institute, Amsterdam University Medical Center, location VU University Medical Center, Amsterdam, the Netherlands; 2 Star-SHL diagnostic centers, Etten-Leur, the Netherlands; 3 Department of Health Sciences, VU University, Amsterdam, the Netherlands; 4 Department of Family Medicine, CAPHRI Care and Public Health Research Institute, Maastricht University, Maastricht, the Netherlands; 5 National lnstitute for Public Health and the Environment (RlVM), Bilthoven, the Netherlands; 6 Department of General Practice, Julius Centrum, University Medical Center Utrecht, Utrecht, the Netherlands

**Keywords:** Respiratory tract infection, Nursing home, Antibiotic prescribing, CRP, Point-of-care testing

## Abstract

**Background:**

Antibiotics are over-prescribed for lower respiratory tract infections (LRTI) in nursing home residents due to diagnostic uncertainty. Inappropriate antibiotic use is undesirable both on patient level, considering their exposure to side effects and drug interactions, and on societal level, given the development of antibiotic resistance. C-reactive protein (CRP) point-of-care testing (POCT) may be a promising diagnostic tool to reduce antibiotic prescribing for LRTI in nursing homes. The UPCARE study will evaluate whether the use of CRP POCT for suspected LRTI is (cost-) effective in reducing antibiotic prescribing in the nursing home setting.

**Methods/design:**

A cluster randomized controlled trial will be conducted in eleven nursing homes in the Netherlands, with the nursing home as the unit of randomization. Residents with suspected LRTI who reside at a psychogeriatric, somatic, or geriatric rehabilitation ward are eligible for study participation. Nursing homes in the intervention group will provide care as usual with the possibility to use CRP POCT, and the control group will provide care as usual without CRP POCT for residents with (suspected) LRTI. Data will be collected from September 2018 for approximately 1.5 year, using case report forms that are integrated in the electronic patient record system. The primary study outcome is antibiotic prescribing for suspected LRTI at index consultation (yes/no).

**Discussion:**

This is the first randomised trial to evaluate the effect of nursing home access to and training in the use of CRP POCT on antibiotic prescribing for LRTI, yielding high-level evidence and contributing to antibiotic stewardship in the nursing home setting. The relatively broad inclusion criteria and the pragmatic study design add to the applicability and generalizability of the study results.

**Trial registration:**

Netherlands Trial Register, Trial NL5054. Registered 29 August 2018.

## Background

Lower respiratory tract infections (LRTI) such as pneumonia are common in nursing homes (NHs) [1]. In Dutch NHs in 2015, the incidence of suspected pneumonia was approximately 200 cases per 1000 residents, with a typical pattern of seasonal variation (i.e. winter peak, summer trough) [2]. NH residents are at increased risk of respiratory infections due to factors typical for this population, such as frailty and comorbidities. Also, the crowded nature of NH residence and frequent nursing staff-resident contact may facilitate the transmission of pathogens [1, 3–5]. The incidence of nursing home-acquired pneumonia (NHAP) can be up to tenfold of the incidence in elderly living in the community [1, 6–10]. Moreover, the severity and prognosis of NHAP is worse compared to pneumonia among elderly living in the community [11–13]. NH LRTI episodes may range from self-limiting viral infections, to severe NHAP requiring hospitalization or causing rapid death [3, 14–16].

An early diagnosis of NHAP enables prompt and appropriate management, decreases the risk of complications and mortality, and reduces overall health care costs [16–18]. Yet, physicians often find it difficult to estimate the severity and potential outcome of the LRTI episode [19, 20]. Moreover, NH residents often have atypical clinical presentation, multi-morbidity, and a diminished ability to recall or describe symptoms (e.g. due to cognitive impairment). Diagnostic tools are often not available (e.g. chest X-ray) or applicable (e.g. sputum culture) in this setting [3, 5, 17, 21, 22]. Overall, diagnostic uncertainty often results in (empirical) antibiotic prescribing to be ‘better safe than sorry’. This attitude towards antibiotic prescribing may be reinforced by external factors, such as (perceived) expectations of patients or family members towards antibiotic prescribing [23, 24].

Antibiotics are among the most commonly prescribed drugs in NHs, however, many of these prescriptions are inappropriate [25]. Inappropriate, or ‘unjustified’, antibiotic prescribing for LRTI in NH or care homes ranges from 25 to 98%, according to studies from different settings and with different criteria for defining inappropriateness [21, 22, 26–29]. Overprescribing of antibiotics has possible negative consequences for the patient, such as drug interactions and side effects [21, 27]. At a societal level, overprescribing of antibiotics contributes to the development of antibiotic resistance, which decreases treatment possibilities for future LRTI [5].

C-reactive protein (CRP) point-of-care testing (POCT) is a promising tool to decrease the diagnostic uncertainty regarding suspected LRTI in the NH setting, and therefore decrease inappropriate antibiotic prescribing. CRP is a dynamic biomarker of the presence and severity of inflammation. CRP increases within four to 6 hours after the onset of an inflammatory reaction as well as rapidly decreases after its resolution (4 to 7 hours half-time, and 19 hours half-life) [30–32]. CRP POCT alongside the clinical signs and symptoms may provide the physician with valuable information for the treatment decision [33]. Studies in the general practice population showed CRP to be the strongest predictor of pneumonia, and that the reliability of the diagnosis improves when CRP is added to the evaluation of clinical signs and symptoms [19, 34]. The introduction of CRP POCT in general practice has resulted in a significant and cost-effective reduction in antibiotic prescribing for LRTI in adults as well as in adults with underlying COPD, without negative consequences for clinical recovery [14, 35–37].

At present, CRP POCT is widely used in general practice in several countries, including the Netherlands. In the NH setting, however, (cost-) effectiveness of CRP POCT on antibiotic prescribing for LRTI has not yet been investigated. Consequently, CRP POCT is not commonly used in this setting. However, CRP-values may also have value in this setting. For instance, there is evidence that the CRP-level at index consultation predicts severity and outcome of pneumonia in the elderly population [38, 39].

This study protocol paper describes the design of a cluster Randomized Controlled Trial (RCT) that investigates whether the use of CRP POCT results in a safe reduction in antibiotic prescribing for NH residents with suspected LRTI. Other questions we aim to address are the extent to which CRP POCT values correlate with A) signs and symptoms in NH patients with suspected LRTI, and B) antibiotic treatment. Also, we will evaluate the cost-effectiveness and cost-benefit of the use of CRP POCT in the NH setting.

## Methods/design

### Study design and population

The UPCARE study design is a cluster RCT, with randomization at NH organization level. This randomization level was chosen because of our pragmatic trial design and in order to avoid spill-over effects. Data collection starts September 2018 and will, based on calculations of expected inclusion rate, last approximately 1.5 year. Eleven NH organizations across the Netherlands will participate in the study. A simple randomization procedure using Microsoft Office Excel 2016 software will be performed by the research team to allocate participating organizations to either the control or intervention group (1:1).

Dutch NHs typically have three types of specialized wards: somatic wards that accommodate physically disabled residents, psychogeriatric wards that accommodate residents with dementia, and geriatric rehabilitation wards. NH admission to one of these wards and the required level of care is determined by a standardized assessment performed by a government agency (“Centrum Indicatiestelling Zorg” (CIZ)). Unique to Dutch NHs is the employment of specialized ‘elderly care physicians’. Other NH prescribers may include physicians with other specializations or general medical training, elderly care physicians in training, and nurse practitioners. Dutch NH medical care typically excludes the use of intravenous drugs, and hospital referrals are limited [40, 41].

The study population comprises NH residents from psychogeriatric, geriatric rehabilitation, or somatic wards, who are newly diagnosed with a ‘suspected LRTI’. Patients are excluded if they receive palliative/terminal care with a restrictive antibiotic policy, if they do not wish to be treated with antibiotics, if they are using antibiotics (currently or in the past week), or if they have an infection other than the suspected LRTI (currently or in the past week).

### Sample size calculation

Based on previous study data [40], we expect 15% less antibiotic prescriptions in the intervention group compared to the control group (i.e. 80% respectively 95%). In order to detect this difference, with 80% power and at a 5% significance level, 146 cases would be required. If we randomize eleven NH organizations with an average number of 400 residents (cluster size) and with an intracluster correlation coefficient of 0.06, the required number of cases is 671 [42].

The expected incidence rate of suspected LRTI is 3.5 cases per 1.000 resident-care weeks in NHs [2]. Based on previous study data, we expect that at most 10% of cases will not meet inclusion criteria and that 75% of eligible cases provide informed consent for study participation. This means that of all LRTI cases, approximately 70% can be included in the study, which translates into an expected 2.4 suspected LRTI per 1.000 resident-care weeks.

ln order to include 671 cases of LRTI in eleven organizations with an average number of 400 residents the study period totals to 1.5 year, with a small margin for potential suboptimal inclusion.

### Intervention

In the intervention group, CRP POCT can be used on-site for residents with suspected LRTI in addition to usual care. The control group provides usual care without the possibility for CRP POCT. Usual care may in some cases include CRP-measurement via laboratory assessment, or, in rare cases, sputum culture or chest radiography.

CRP-measurement via laboratory assessment differs from CRP POCT with regard to the type of blood collection (venipuncture respectively finger prick), location (the blood is taken to an external lab respectively the measurement is performed on-site), time-to-results (hours-days versus minutes), and potential frequency of measurements (once-twice a week on average versus 24/7).

During the trial, physicians in the intervention group decide on whether or not to use CRP POCT and, if performed, they consider the results alongside clinical features of the patient in their prescribing decision.

Prior to study commencement, the intervention group will receive two training sessions: 1) a medical training session and 2) a technical POCT training session.

#### Medical training session

Members of the research team will provide a medical training for medical doctors and nurse practitioners in the intervention group on the use and interpretation of CRP POCT for the diagnosis of LRTI. The contents of the training are based on the LRTI guideline for the NH setting (of the Dutch Association of Elderly Care Physicians and Social Geriatricians), and on extensive literature research. Topics include characteristics of the CRP POCT instrument (e.g. validity, reliability, and limitations), evidence and lessons learnt from the use of CRP POCT in general practice, and instructions specific to the NH setting. Specific instructions include the use of cut-off-values for antibiotic prescribing that are included in the LRTI guideline, i.e. a lower limit of 20 mg/L and an upper limit of 60 mg/L. The latter is different from the cut-off-value in the general practice population (i.e. 100 mg/L), and was based on a NH study that showed adequate discriminatory power for distinguishing pneumonia with this CRP-value [33].

#### Technical POCT training session

The POCT expert team from a non-commercial, EU accredited laboratory (Saltro diagnostic center, Utrecht) will provide the technical instructions to the intervention group of medical doctors and nurses who will perform CRP POCT during the trial. In each NH organization, a ‘trainer’ is appointed, who will train new employees during the study period. The technical training takes place after the medical training, within a period of 2 months. After the technical training until study commencement, the intervention group will have a run-in period to get used to CRP POCT in routine practice, varying from 1 week to 3 months per organization. The POCT expert team provides technical assistance during the trial and monitors quality throughout the study period.

#### Technical features of CRP POCT

The CRP POCT instrument that is used in this study (QuikRead go®, Orion Diagnostica Oy) has been shown to have adequate analytical accuracy and agreement with laboratory measurements [43, 44]. The analyzer has a built-in self-check procedure that secures its correct use and correct results. In case of errors, the display will show a specific error notification.

The test principle relies on immunoturbidic measurement of turbidity changes in the sample due to the reaction of CRP with the reagent, i.e. monoclonal antihuman CRP F (ab) 2 fragments-coated microparticles.

The blood sample obtained by finger-prick, using a 20 μl capillary tube, is dispensed into the cuvette, which hemolyzes the blood cells in the sample. Next, the cuvette is placed into the analyzer, which measures the hematocrit level before adding the reagent from the cap into the cuvette. With the reagent added, the microparticles bind to the CRP in the sample. The turbidity of the sample is then calculated using calibration information and with correction for the hematocrit level. In total, the CRP POCT measurement takes 2 to 4 min.

### Data collection

The period of data collection for each participant is 3 weeks. In this period, three case report forms (CRFs) are to be completed by the physician: at index consultation (T0), and at one (T1) and three (T2) weeks after index consultation. The CRFs are integrated into the electronic patient record system: eligibility criteria appear in an eligibility form if physicians diagnose a suspected LRTI. If eligible, CRFs are available electronically to be completed at due time points.

**Fig. 1:**
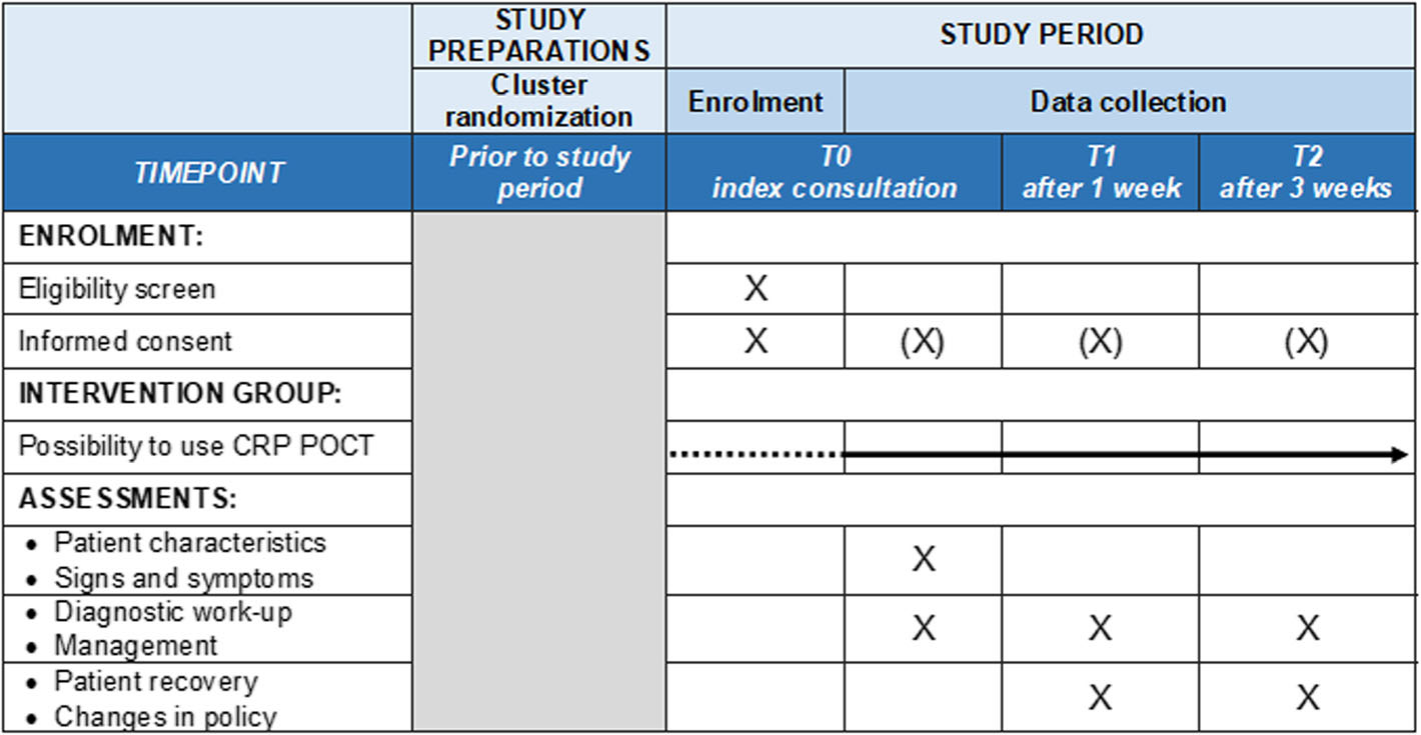
schedule of patient enrollment, intervention, and data collection

The T0 CRF contains questions on patient characteristics (principal diagnosis at NH admission, comorbid conditions, use of immunosuppressive medication, and recent surgery), signs and symptoms, performed diagnostics (CRP POCT and/or other), and antibiotic prescribing (yes/no, type). The T1 and T2 CRF include follow-up on patient recovery, changes in policy (additional diagnostics performed, hospital referral, and treatment changes). In addition, pharmacy data will be collected on total antibiotic prescribing in the NH during the study period. Figure 1 depicts the timeline of patient enrollment, intervention, and data collection.

### Informed consent procedure

The informed consent procedure consists of two steps:

1. Written information is provided to either the patient or representative (depending on competence status, as judged by the physician), prior to study commencement or upon NH admission. At this time and throughout the study period, the patient/representative is given the opportunity to opt-out. Opting-out is registered within the electronic patient record, which deactivates potential eligibility notifications*.* If opted-out, step two will not be initiated.
2. In case of a suspected LRTI, the physician contacts the patient/representative to ask for consent, which includes the opportunity to ask questions. In both groups, consent is asked for data collection. In the intervention group, consent is additionally asked for performing CRP POCT. An exceptional case is the situation in which the patient or the representative is not able to provide consent at the time of the diagnosis (e.g. if the patient is too ill or if the representative is not present): in that case, a physician can use CRP POCT as part of usual care if this is considered to support proper management. Consent is asked in retrospect (i.e. deferred consent), as soon as possible and at an appropriate moment, for data collection and potential future CRP POCT use. After the physician confirms within the CRF that the informed consent form is signed, data from the CRF are automatically sent to the research team in pseudonymized form via a secure web-portal.

### Outcomes

The primary study outcome is antibiotic prescribing for suspected LRTI at index consultation (yes/no). Secondary study outcomes include physician-reported recovery at one and 3 weeks after index consultation, use of additional diagnostics within 3 weeks after the index consultation (including repeated CRP measures), changes in treatment policy within 3 weeks after the index consultation, hospitalization, complications, (all-cause) mortality, and total antibiotic prescribing on the NH level.

Costs that will be included in the economic evaluation (health care perspective) are related to the use of CRP POCT, prescription of antibiotics, consultation by physicians in the NH, additional diagnostic tests, hospital admissions and other health care utilization for LRTI and complications of LRTI treatment. Costs will be measured from the CRFs and valued using the guidelines of the National Health Care Institute [45].

### Data analysis

The primary analysis will be intention-to-treat and will assess the effect of CRP POCT on antibiotic prescribing for suspected LRTI at index consultation. A three-level logistic regression model will be used to account for variation at the NH/physician/patient level. If there is no indication of random effects at the physician level, the model will be reduced to a two-level model. Multilevel regression modeling will similarly be used to compare secondary study outcomes between the two groups (linear or logistic, as appropriate). A second-order penalized quasi-likelihood estimation procedure will be applied.

Pharmacy data on total antibiotic prescribing will be explored descriptively, in order to describe the potential impact of adjusted antibiotic prescribing for LRTI on total antibiotic prescribing within intervention NHs, compared to control NHs. Total antibiotic prescriptions will be expressed per 1000 residents per year.

Data of patients in the intervention group will be used to explore potential relations between CRP POCT values and; 1) signs/symptoms in NH patients with suspected LRTI, and 2) antibiotic treatment. Multiple linear or logistic (as appropriate) regression modeling will be performed.

### Cost-effectiveness and cost-benefit analyses

The cost-effectiveness analysis includes the percentage of antibiotic prescribing as outcome. A cost-benefit analysis, in which the reduction in antibiotic prescribing will be expressed in monetary terms, will also be performed. Missing data will be imputed in the cost-effectiveness analysis using multiple imputation techniques. Fully Conditional Specification and Predictive Mean Matching will be used to create ten complete data sets. Pooled estimates will be calculated according to Rubin’s rules [46]. We will calculate the mean differences for total and disaggregated costs and perform seemingly unrelated regression analyses, correcting for baseline characteristics and taking into account possible correlations between costs and effects. An incremental cost-effectiveness ratio will be calculated, with a corresponding cost-effectiveness plane. The cost difference and incremental cost-effectiveness ratio will be Bootstrapped with 5000 replications. The probability of cost-effectiveness at different values of willingness-to-pay will be estimated and presented on a cost-effectiveness acceptability curve. We will conduct sensitivity analyses on uncertain parameters to evaluate the robustness of the results.

## Discussion

This protocol paper describes the design of a cluster RCT to assess the effect of CRP POCT on antibiotic prescribing for LRTI in NHs. This is, to our best knowledge, the first large RCT to evaluate this topic in the NH setting. With this study, we aim to contribute to antibiotic stewardship efforts in the NH setting.

### Reflection on study design

#### Study population

We use broad inclusion criteria for the study population, for example we include patients from somatic, geriatric rehabilitation, and psychogeriatric wards. Research in the psychogeriatric population may be challenging, for instance with regard to obtaining informed consent. However, results of this study are especially important for this population as quick diagnosis and treatment initiation can be challenging (i.e. difficult clinical assessment) but essential given the vulnerability of this population [5, 16, 17, 21].

#### Control group

During the study we remain vigilant towards potential post-randomization recruitment bias: the control group might gradually or throughout the trial be less inclined to recruit patients, given the absence of the intervention [14, 20]. We anticipate the need for incentives especially in the control group during the trial. Another phenomenon that may arise in the control group is the Hawthorne effect, that is, a shift towards more rational antibiotic prescribing because of the physician’s awareness of being observed [29, 47].

#### Data collection

An anticipated strength of the study is the data collection method. The integration of the research tool in the electronic patient file ensures that data are collected in an efficient way. In addition, the use of automatic reminders and other technical support reduce the risk of missing data.

#### Informed consent procedure

Reasons for exercising a deferred consent procedure stem from parallels seen in emergency research to this study, which we consider in emergency situations and in case the representative is unavailable [48, 49].

In case of certain emergency situations, CRP POCT could directly benefit patient care. For instance, CRP POCT might provide the physician with valuable information for the differential diagnosis between LRTI and congestive heart failure. Another consideration in asking consent during an emergency situation is that it might conflict with sufficient comprehension of study participation and with the principle of “evidencing a choice” [49].

Another situation that warrants deferred consent arises when the representative of an incapacitated resident is not readily available at index consultation. In that case, a requirement for prior consent could interfere with the time span within which CRP POCT is still worthwhile. Consequently, this subpopulation is unnecessarily disadvantaged in diagnostic possibilities - assuming the added value of CRP POCT that is seen in general practice and considering the higher burden of venipuncture compared to finger prick if CRP would instead be determined by laboratory assessment. Also, selection bias may appear if this subpopulation is more often excluded from the study because of difficulty in obtaining consent.

The alternative to ask consent pre-emptively was considered, however, this would require burdening a disproportionate number of residents with the question of consent, compared to those becoming eligible for participation. Moreover, as the period between consent and study participation could be lengthy the resident may not remember the choice for and extent of study participation.

### Reflection on study context

#### Antibiotic stewardship in NHs

In the Netherlands in recent years, antimicrobial resistance in different settings such as NHs has gained a more prominent place on the research and public health agenda. Antibiotic stewardship efforts are widely supported and developed for the NH setting by different parties. During the UPCARE study, it is important to monitor such activities as they might influence the primary outcome.

#### LRTI guideline for the NH setting

Around the start of data collection, a LRTI guideline was published for the NH setting by the Dutch Association of Elderly Care Physicians and Social Geriatricians. In this guideline, physicians are instructed to assess the CRP-level for patients who are moderately ill and who have certain clinical signs and symptoms that do not indicate LRTI unambiguously. In the guideline, CRP POCT is not specifically advised for CRP-measurement, as evidence for its (cost-) effectiveness is currently insufficient. With the UPCARE study we aim to address this knowledge gap.

#### Pragmatic trial design

We advise physicians and nurses in the intervention group on the possible use of CRP POCT and support them in this matter, but we do not use strict protocols on the use and interpretation of CRP POCT; physicians remain in charge of their diagnostic work-up and management decisions. This pragmatic design enables us to observe an effect that reflects daily practice. This increases the chance that our findings will be generalizable and widely applicable [14, 20]. A potential pitfall of this approach is that the medical training session and other study preparations might not sufficiently incite behavioural change. Essential to the potentiality of a positive effect of the intervention is that physicians learn to trust CRP POCT findings and subsequently use these findings to adjust management when appropriate [20]. However, the use of CRP-measurements in general is not new and results from general practice are encouraging. Furthermore, all NHs will have a run-in period before study commencement to familiarise themselves with the use of CRP POCT (device and results). We will perform a process evaluation to explore the extent to which the intervention has been successfully implemented and used.

## Conclusion

This is the first large RCT to evaluate CRP POCT for suspected LRTI in the NH setting. The broad inclusion criteria and pragmatic study design add to the applicability and generalizability of the study results. With this study we aim to contribute to antibiotic stewardship efforts in the NH setting.

## Data Availability

The datasets generated and/or analysed during the current study will be deposited in the repository DANS (EASY) after publication of the research results, within a maximum of 9 months post study termination. The dataset(s) involved will be anonymised/pseudonymised and can be accessed under restrictions.

## Abbreviations

CRF: Case Record Form
CRP POCT: C-Reactive Protein Point-Of-Care Test(ing)
LRTI: Lower Respiratory Tract Infection
NH: Nursing Home
NHAP: Nursing Home-Acquired Pneumonia
RCT: Randomized Controlled Trial
